# UBE2D3 gene overexpression increases radiosensitivity of EC109 esophageal cancer cells *in vitro* and *in vivo*

**DOI:** 10.18632/oncotarget.8869

**Published:** 2016-04-20

**Authors:** Xiaojia Gao, Wenbo Wang, Hui Yang, Lin Wu, Zhongshi He, Shuliang Zhou, Hong Zhao, Zhenming Fu, Fuxiang Zhou, Yunfeng Zhou

**Affiliations:** ^1^ Hubei Cancer Clinical Study Center, Hubei Key Laboratory of Tumor Biological Behaviors, Wuhan, China; ^2^ Department of Radiation and Medical Oncology, Zhongnan Hospital of Wuhan University, Wuhan, China; ^3^ Hubei Cancer Clinical Study Center, Zhongnan Hospital of Wuhan University, Wuhan, China

**Keywords:** UBE2D3, ubiquitin, hTERT, radiosensitivity, esophageal cancer

## Abstract

Ubiquitin-conjugating enzyme E2D3 (UBE2D3), a key component in ubiquitin (Ub) proteasome system, plays a crucial role in tumorigenesis. We previously found that it is bound to hTERT, and UBE2D3 could attenuate radiosensitivity of human breast cancer cells. Here we investigated a contributing role of UBE2D3 in radiosensitivity of esophageal squamous carcinoma. We demonstrated that the overexpression of UBE2D3 in esophageal squamous carcinoma cells (EC109) resulted in prolonged G1 phase and shortened G2/M phase after irradiation. UBE2D3 overexpression also decreased length of telomere and activity of telomerase. In addition, the overexpression of UBE2D3 increased mRNA expression but decreased protein levels of hTERT in both *vitro* and *vivo* systems. Compared with untreated cells, the treatment of UBE2D3 overexpressing cells with the specific proteasome inhibitor (MG132) could up-regulate hTERT. MG132 treatment of UBE2D3 overexpressed cells caused a clear and dramatic increase in the amount of ubiquitinated hTERT species. These findings indicate that UBE2D3 enhances radiosensitivity of EC109 cells by degradating hTERT through the ubiquitin proteolysis pathway.

## INTRODUCTION

Esophageal cancer (EsC) is one of the most common malignant tumors in China [1]. Radiotherapy is one of the main treatments to reduce local recurrence and improve overall survival of EsC. The current overall 5-year survival of EsC is only about 16.9~20.9% [1, 2]. Therefore, it is of importance to improve the efficacy of radiotherapy of EsC. We previously documented that radiosensitivity was negatively associated with telomerase activity [3–7]. Telomerase comprises three major components: telomerase RNA, telomerase-associated protein and the catalytic protein subunit of telomerase (hTERT) [8]. Our early study showed that UBE2D3 interacted with hTERT and co-localized with it in cell nucleus [9]. UBE2D3 was negatively correlated with hTERT expression in EsC tissues [10].

UBE2D3, also named UbcH5c, is a member of ubiquitin-conjugating enzyme (E2) family, which is a key component in ubiquitin (Ub) proteasome system (UPS) [11]. Ubiquitin-dependent proteolysis by the 26S proteasome plays a pivotal role in tumorigenesis [12]. In this pathway, E2, which is including UBE2D3, together with ubiquitin ligase (E3), transfers ubiquitin to the specific substrate protein(s) [9]; Polyubiquitinated proteins are recognized by the 26S proteasome and rapidly degraded [13]. It has been shown that the expression of UBE2D3 was extremely low in all of the cancerous cell lines including esophageal cancer cell line but not in normal tissues [14]. We previously found that the inhibition of UBE2D3 could decrease radiosensitivity of MCF-7 cells by upregulating hTERT expression and activity [9]. In addition, we found that UBE2D3 was negatively correlated with hTERT expression and was a positive prognostic factor for EsC [10]. Although hTERT expression has been shown to be negatively associated with radiosensitivity of various of cancers including EsC [15, 16], little is known about the role of UBE2D3 in radiosensitivity of EsC.

Therefore, in this study, we examined the impact of UBE2D3 on radiosensitivity of esophageal squamous carcinoma cells. First, we constructed stable UBE2D3-overexpressed EC109 cell line; Second, we confirmed the radiosensitivity by clonogenic assay; Third, we explored the mechanism by flow cytometry, PCR, western blotting, PCR-ELISA, immunofluorescence and immunoprecipitation assay; Last, we reproduced the *in vitro* result in nude mice by immunohistochemical analysis.

## RESULTS

### Overexpression of UBE2D3 enhanced radiosensitivity of EC109 cells by modifying cell cycle after IR

**Figure 1:**
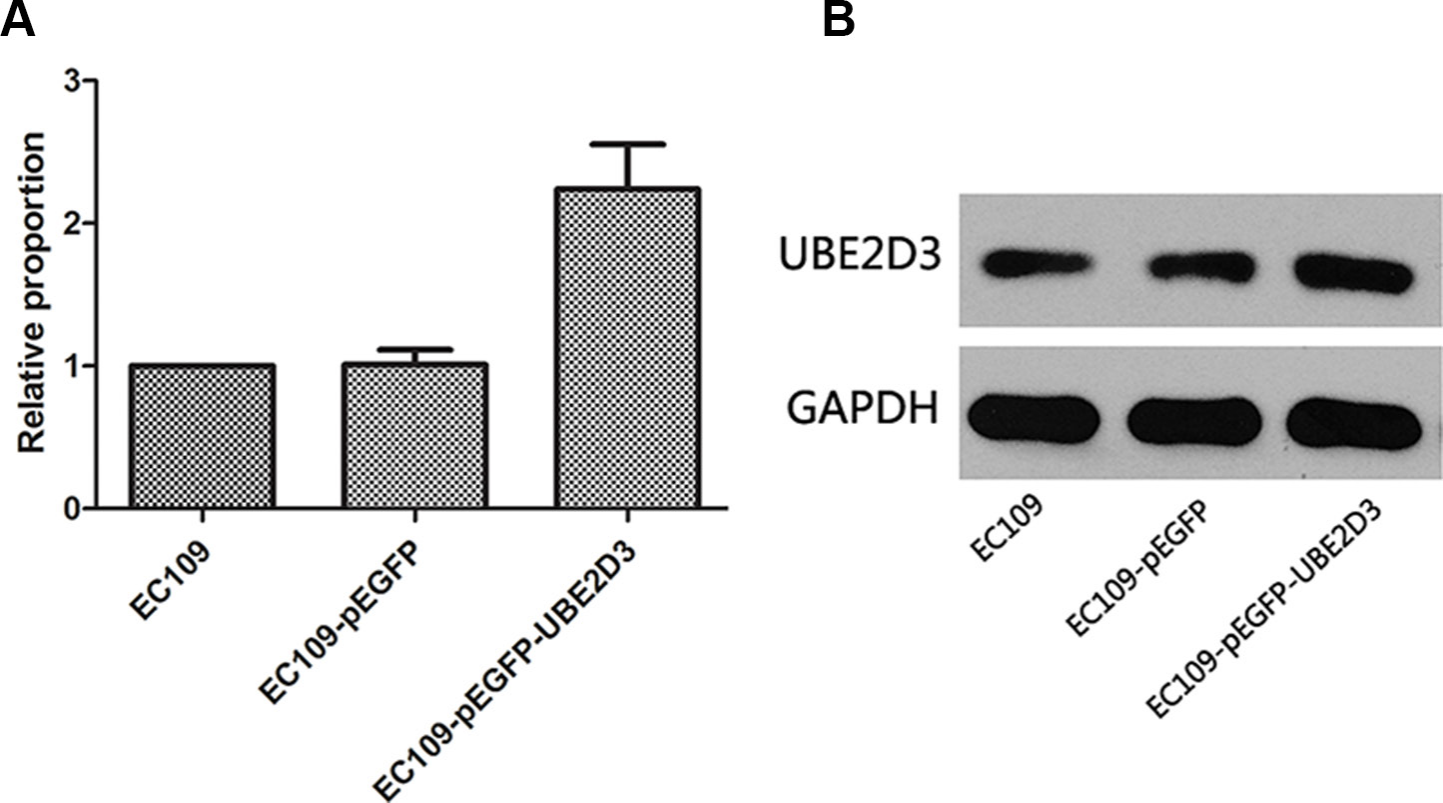
Verification of UBE2D3 overexpression by PCR and western blotting (**A**) Relative to EC109 cells, the mRNA expression level in EC109-pEGFP-UBE2D3 cells was 2.239 (*P* = 0.024, *t* = 3.712), and in EC109-pEGFP cells was 1.010 (*P* = 0.936, *t* = 0.089). (**B**) Relative to EC109 cells, the protein expression level in EC109-pEGFP-UBE2D3 cells was 1.362 (*P* = 0.004, *t* = 5.816), and in EC109-pEGFP cells was 0.888 (*P* = 0.241, *t* = 1.377).

The mRNA and protein expression of UBE2D3 was determined in EC109 cells transfected with the pEGFP-UBE2D3 plasmid (Figure 1A and 1B). Compared with the untransfected cells, there was a significant increase (*P* = 0.024, *t* = 3.712; *P* = 0.004, *t* = 5.816) in UBE2D3 expression in the transfected cells, UBE2D3 expression was not affected (*P* = 0.936, *t* = 0.089; *P* = 0.241, *t* = 1.377) in EC109 cells transfected with the control plasmid (pEGFP).

**Figure 2:**
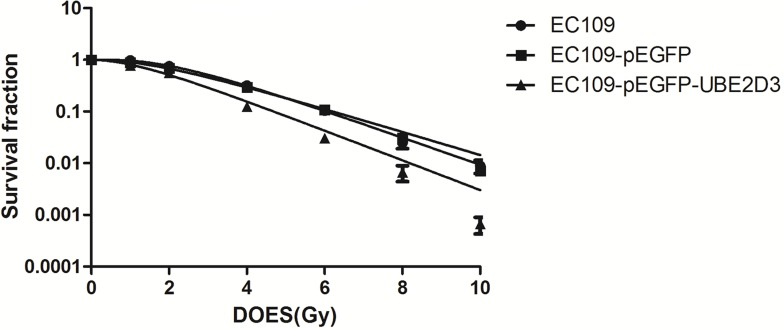
Effects of UBE2D3 overexpression on the radiosensitivity in EC109 cells Each group of cells was irradiated with 0, 1, 2, 4, 6, 8 and 10 Gy respectively. After 2 weeks incubation, the colonies were fixed and stained. The data were fit into multitarget-single hit models to assess the radiosensitivity of cells. At each dose point, surviving fraction of EC109-pEGFP-UBE2D3 cells was lower than of EC109 cells, The similar result was found in EC109-pEGFP cells. SF2 of EC109 cells, EC109-pEGFP cells and EC109-pEGFP-UBE2D3 cells was 0.755 ± 0.162, 0.731 ± 0.216 and 0.486 ± 0.070, respectively. Relative to EC109 cells, EC109-pEGFP-UBE2D3 cells was more sensitivity to X-ray (*P* = 0.008, *t* = 3.672), the sensitivity to X-ray of EC109-pEGFP cells was similar to that of EC109 cells (*P* = 0.846, *t* = 0.201). Each experiment was done at least three times in triplicate wells.

**Figure 3:**
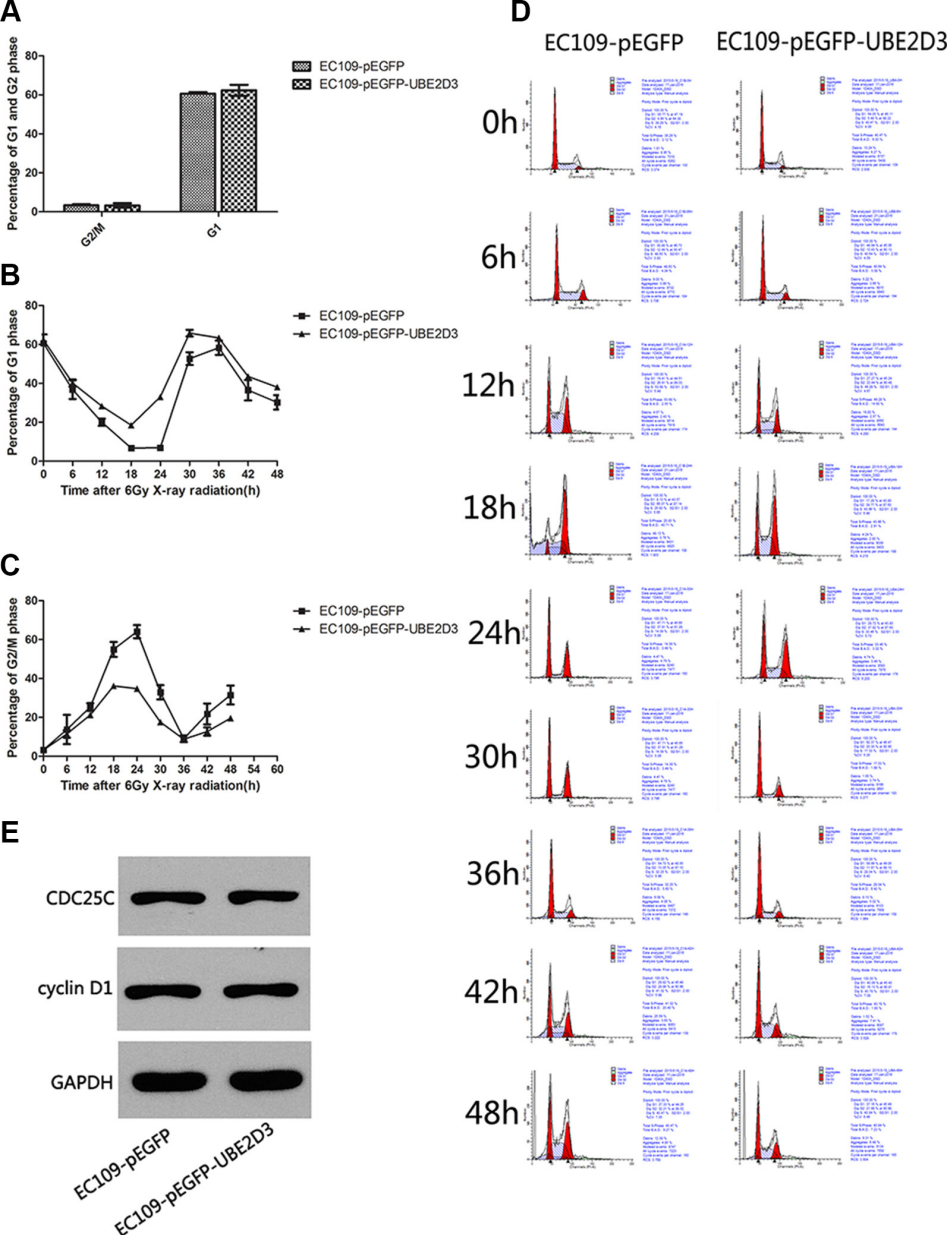
Effects of UBE2D3 overexpression on the cell cycle with or without IR in EC109 cells (**A**) Before X-ray treatment, cell cycle was detected by flow cytometry. In EC109-pEGFP cells and EC109-pEGFP-UBE2D3 cells, the proportion of G2/M phase was 3.323 ± 0.895 and 3.247 ± 1.165, respectively (*P* = 0.933, *t* = 0.090). The proportion of G1 phase was 60.640 ± 1.337 and 62.383 ± 2.788, respectively (*P* = 0.404, *t* = 0.977). (**B**) After 6 Gy X-ray treatment, cell cycle was detected each 6 hours, the percentage of G1 phase in EC109-pEGFP-UBE2D3 cells was obviously higher than that in EC109-pEGFP cells. However, the Figure 3C showed that the percentage of G2/M phase in EC109-pEGFP-UBE2D3 cells was obviously lower than that in EC109-pEGFP cells. (**D**) Cell cycle was evaluated by flow cytometry. (**E**) Western blotting analysis showing the cell cycle check point proteins of CDC25C and cyclin D1 expression level had no significant difference between the two kinds of cells. Experiments were repeated 3 times with similar results.

In clonogenic assay, we used multitarget-single hit models to assess the radiosensitivity (Figure 2). Surviving fraction after 2 Gy X-ray iradiation (SF2) indicated that overexpression of UBE2D3 enhanced radiosensitivity in EC109 cells compared to EC109-pEGFP cells and EC109 cells (*P* = 0.042, *t* = 2.421; *P* = 0.008, *t* = 3.672). There waslittle difference in the cell cycle between these cell lines. (Figure 3A). After 6 Gy X-ray IR, G1 phase arrest was prolonged in UBE2D3-overexpressed cells and G2/M phase was shortened (Figure 3B and 3C). Western blot was used to verify the expression abundance of those check point proteins to test their effect on cell cycle arrest (Figure 3E). There were little differences in the levels of these proteins between the two groups.

### UBE2D3-induced cell cycle arrest is mediated by ATM/ATR-Chk2 pathway

**Figure 4:**
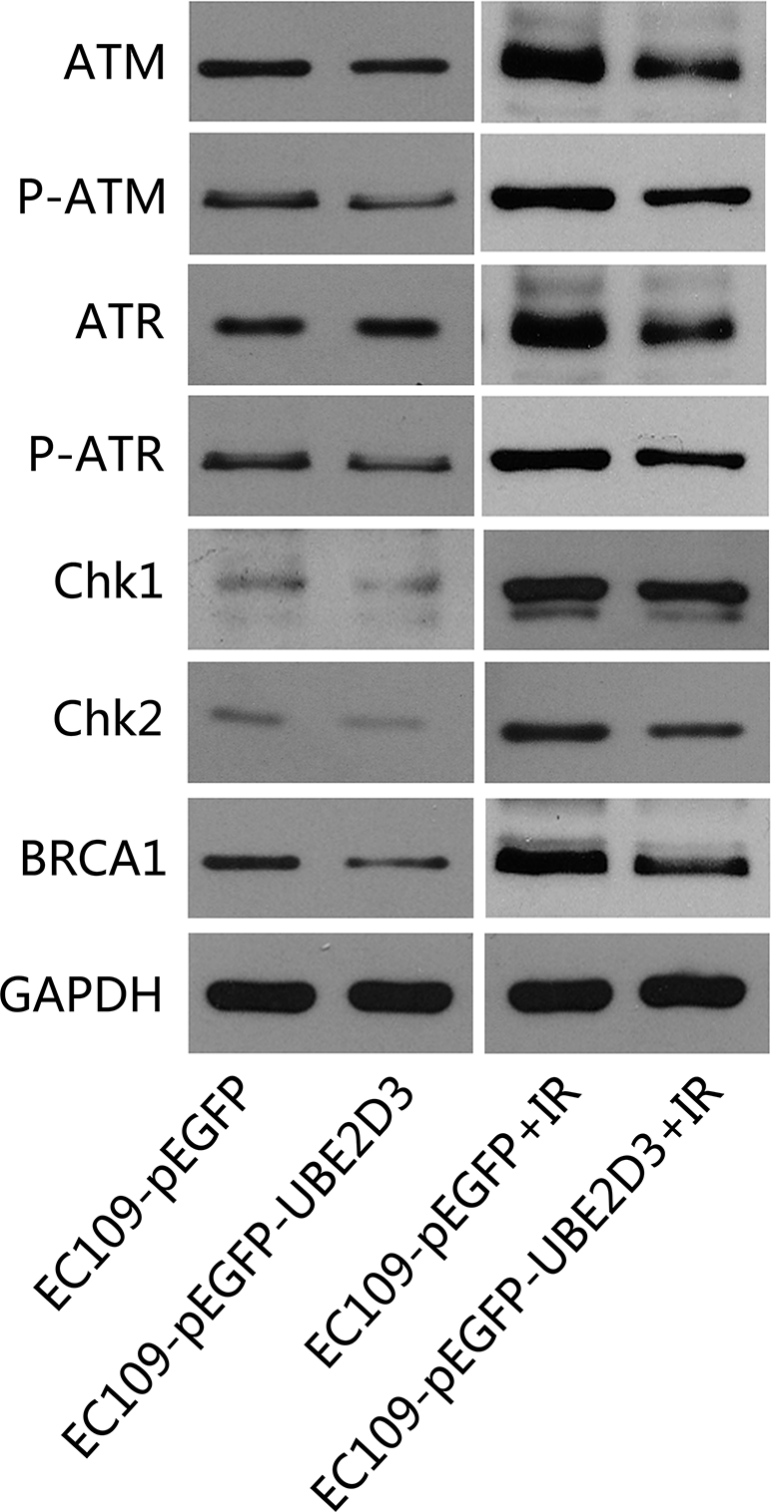
UBE2D3 overexpression decreased DNA damage related proteins after IR Proteins were collected after 24 hours of 10 Gy X-ray treatment, relative proteins expression about DNA damage response were lower in UBE2D3-overexpressed cells. But no obvious difference between the two groups was observed without X-ray disposing. Experiments were repeated 3 times with similar results.

We also evaluated the impact of UBE2D3 on the expression of the DNA damage response proteins. As shown in Figure 4, the DNA damage response proteins (ATM, P-ATM, ATR, P-ATR, CHK1, CHK2 and BRCA1) were significantly downregulated in UBE2D3-overexpressed cells after IR. In contrast, there was little difference between the two groups was observed without IR.

### UBE2D3 overexpression increased DNA damage foci induced by IR

**Figure 5:**
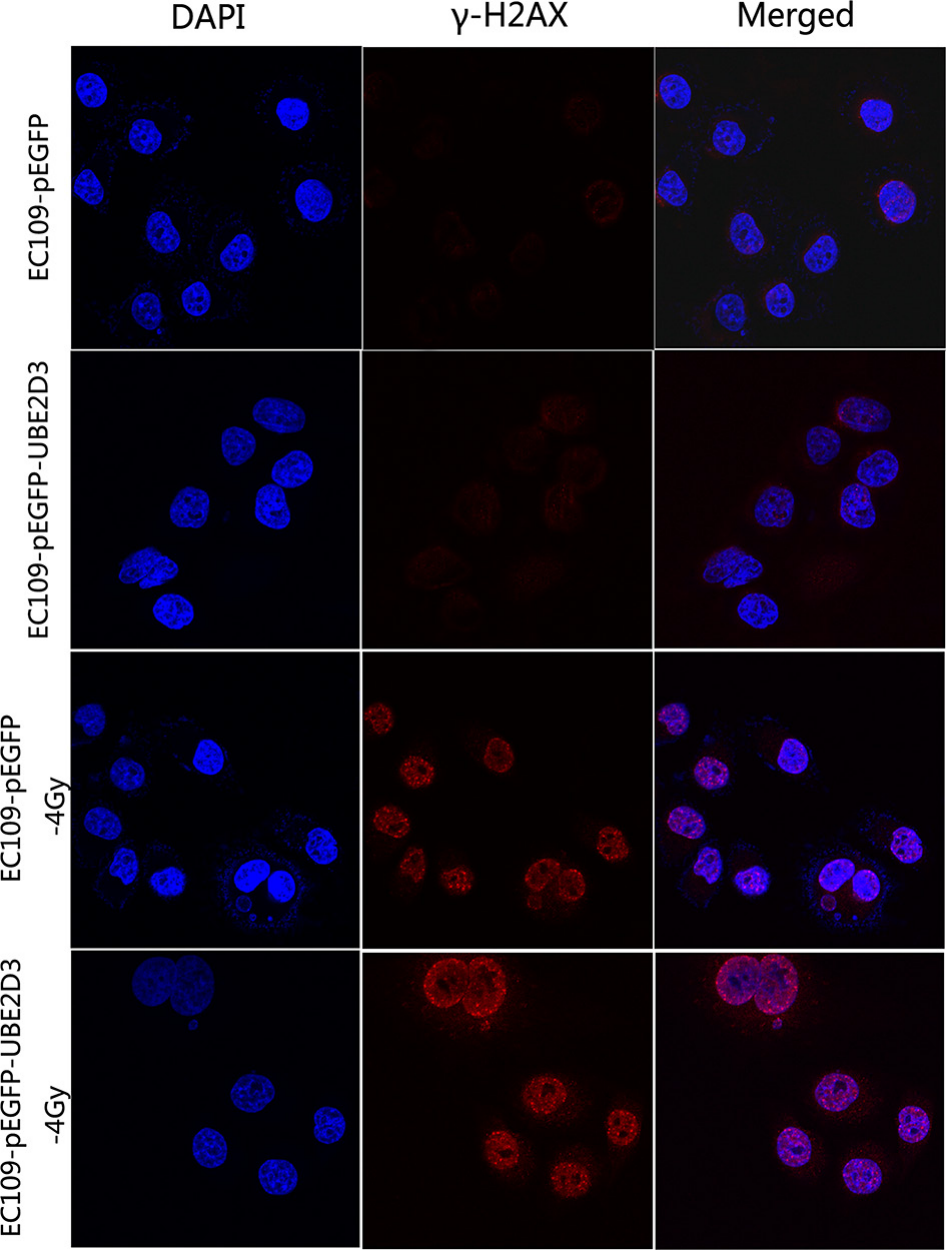
UBE2D3 overexpression inhibited repair of DNA damage induced by IR The cells were exposed to 4 Gy and incubated for 1 h. Results from three representative images for damaged foci are shown. Images shown in the last column were produced by merging all two channels. DNA damage foci were similar between the two groups without irradiation, but enhanced more obviously in UBE2D3-overexpressed cells after irradiation. Cells were enlarged 200 times by microscopy.

The immunofluorescence results showed that the level of γ-H2AX (a DNA damage marker) was little difference between the two groups without IR; However, the X-rays treatment of UBE2D3 overexpressing cells led to an enhanced DNA damage foci (Figure 5).

### Overexpressed UBE2D3 decreased length of telomere and activity of telomerase

**Figure 6:**
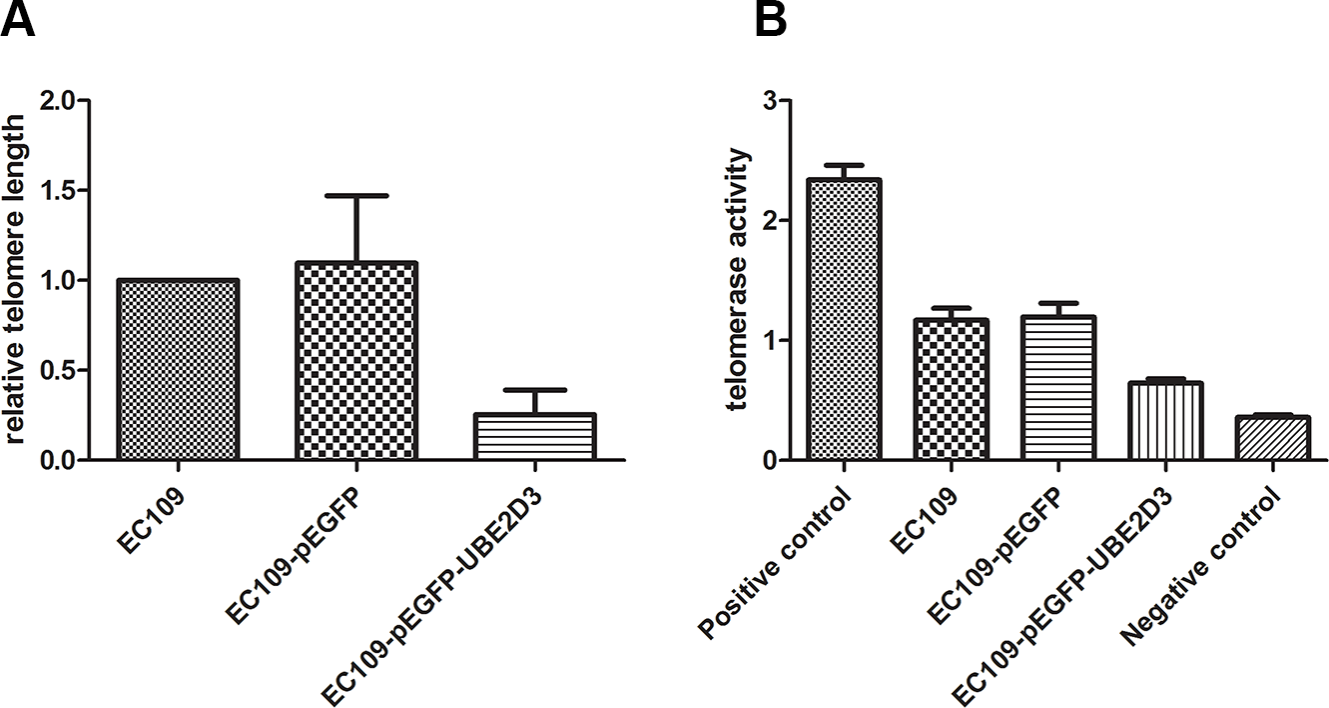
UBE2D3 overexpression shortened telomere length and decreased telomerase activity (**A**) As to EC109 cells, relative telomere length of EC109-pEGFP-UBE2D3 cells was shorter than that of EC109 cells (*P* = 0.002, *t* = 5.463), while no obvious difference of relative telomere length (**B**) was observed between EC109-pEGFP cells and EC109 cells (*P* = 0.817, *t* = 0.253).

To confirm the DNA damage repair capacity which correlates with telomere length, we examined relative telomere length by RT-PCR. As shown in Figure 6A, high contents of UBE2D3 in EC109 cells suppressed the extension of telomere (*P* = 0.002, *t* = 5.463). In addition, telomerase activity decreased significantly after UBE2D3 over expressed (Figure 6B) (*P* = 0.000, *t* = 8.466).

### hTERT was degraded by ubiquitin proteasome pathway

**Figure 7:**
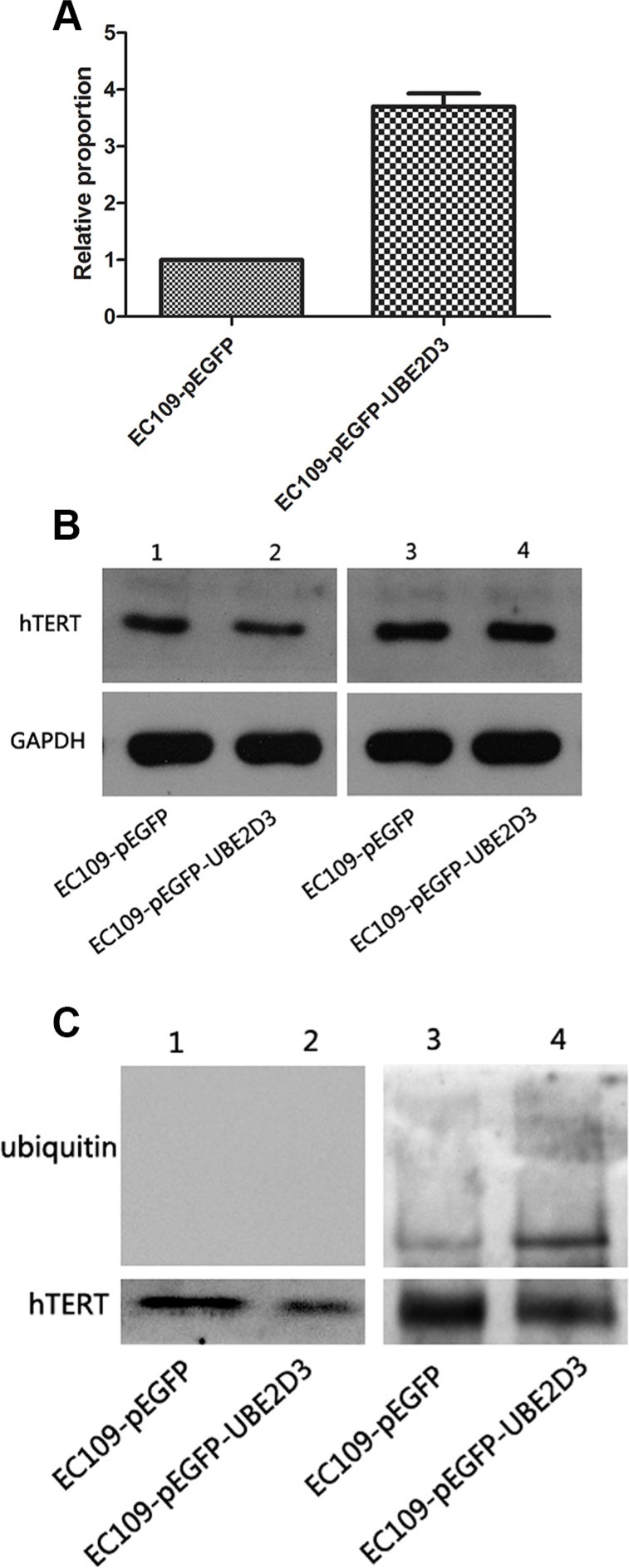
hTERT was degraded by the proteasome pathway mediated by UBE2D3 (**A**) mRNA of hTERT in EC109-pEGFP-UBE2D3 cells was much higher than that in EC109-pEGFP cells (*P* = 0.000, *t* = 28.974) (**B**) Line 1 and 2 were tested before MG132 treatment, result showed that the up-regulation of UBE2D3 decreased the expression of hTERT. Line 3 and 4 were tested after 2 hours of MG132 desposed, aboundance of hTERT in EC109-pEGFP-UBE2D3 cells almost reached the same level to that in EC109-pEGFP cells. (**C**) hTERT protein was obtained by co-immunoprecipitation assay, and anti-ubiquitin antibody was used in immunoblotting to value the ubiquitination of hTERT. Almost discovery nothing in line 1 and 2 without MG132 treatment, but after MG132 treatment, the ubiquitin level in EC109-pEGFP-UBE2D3 cells was dramatic higher than that in EC109-pEGFP cells. Indicated that ubiquitined hTERT was up-regulated by UBE2D3 overexpressing.

Telomere is maintained by telomerase [17], hTERT, as the telomerase subunit, plays an important role in this process. mRNA of hTERT was significantly increased after UBE2D3 overexpression (Figure 7A) (*P* = 0.000, *t* = 28.974), while protein abundance decreased significantly (Figure 7B, line1 and 2) in this study. To explore the primary reason for the phenomenon, proteasome inhibitor MG132 (10 μM) was used by 2 hours followed by western blot (Figure 7B, line 3 and 4), Figure 7B showed that abundance of hTERT didn't change obviously before and after the inhibitor treatment in control group (line 1 and 3), but significantly increased in UBE2D3 over-expressed cells than before (line 2 and 4) and content of protein hTERT was similar between two groups after MG132 used (line 3 and 4). The total hTERT protein in the cells was obtained by using the immunoprecipitation method, which followed by mimmunoblotting with anti-ubiquitin antibody to investigate whether UBE2D3 contributes to the ubiquitination of hTERT *in vitro*. There was no any ubiquitin change for those without using MG132 (Figure 7C, line 1 and 2); While two groups can be detected the existence of ubiquitin after MG132 deposed, and the content of ubquitin in UBE2D3 over-expressed cells was much higher than that in control group (Figure 7C, line 3 and 4).

### Tumor growth slowed down *in vivo*

**Figure 8:**
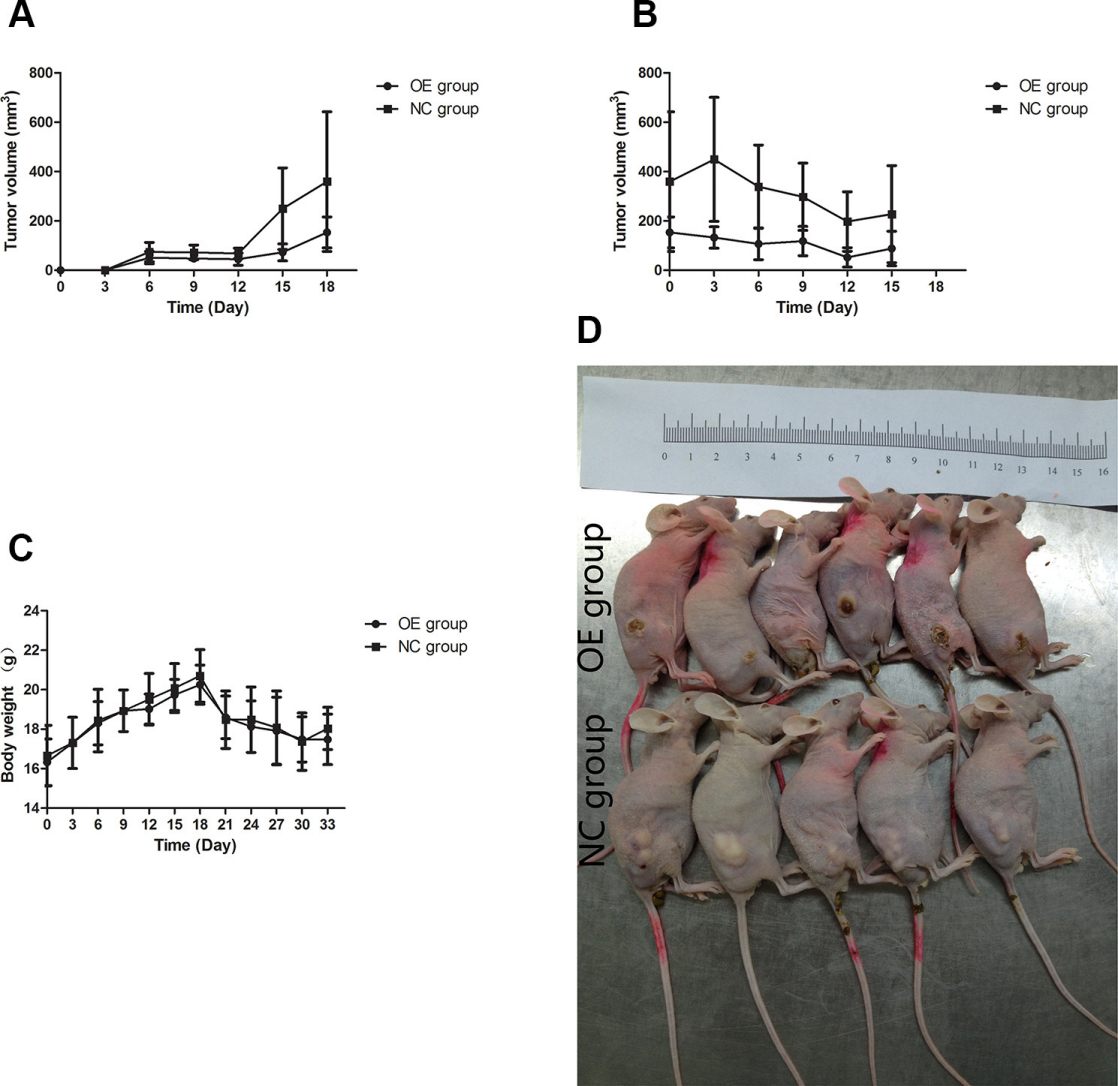
Effects of UBE2D3 overexpression on tumours in nude mice (**A**) EC109-pEGFP cells or EC109-pEGFP-UBE2D3 cells were subcutaneously injected into the right dorsal leg of nude mice, which were named as NC group and OE group respectively. Longest diameter “a” and the shortest diameter “b” of tumors were measured every 3 days, tumor volume (in mm^3^) = a × b^2^ × 0.5. It could be observed that UBE2D3 up-regulation could inhibit tumor growth. (**B**) When the volume of tumors reached 0.5 to 1.0 cm in diameter (around 20 days post injection). The mice was exposed to 10 Gy X-ray once every 6 days for a total of two exposures, it could be observed that tumors in OE group were even disappeared and smaller than that in NC group. (**C**) The body weight of mice was measured every 3 days, no significant difference was found between the two groups. Indicated that the effects of nutrition on tumor growth can be excluded. (**D**) Animals were sacrificed by cervical dislocation, before tumor tissues were excised, mice were arranged together to take photos.

We investigated the *in vivo* impact of UBE2D3 expression on the tumorigenicity and radiation sensitivity of EsC cells by using a mouse model. The tumor volumes of mice in OE group were smaller than those of mice in NC group (Figure 8A). By X-rays treating, difference of tumor volume between the two groups has been expanded (Figure 8B). But no significant difference in body weight of nude mice have been observed (Figure 8C).

### hTERT was down regulated *in vivo*

**Figure 9:**
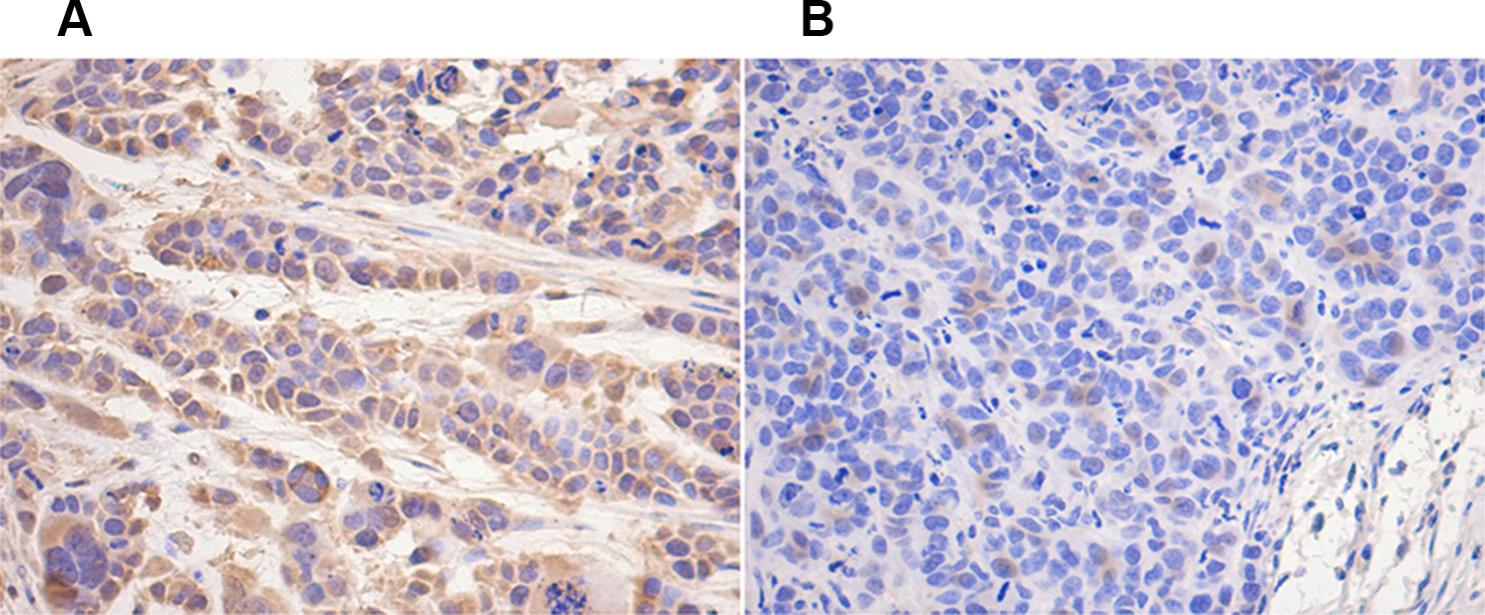
hTERT decreased *in vivo* by UBE2D3 overexpression Immunohistochemical staining of human telomerase reverse transcriptase (hTERT) protein in tumor tissues on nude mice. hTERT expression in OE group (**B**) was lower than that in NC group (**A**). Cells were enlarged 200 times by microscopy.

Immunohistochemical result showed that the expression of hTERT in the tissue of OE group was lower than that in NC group (Figure 9).

## DISCUSSION

In this study, we demonstrated that UBE2D3 overexpression could increase radiosensitivity of EC109 cells by degradating hTERT, a key factor associated with radiosensitivity of tumor cells [3, 6, 15, 16]. The inhibition of UBE2D3 could increase the expression of cell cycle checkpoint protein cyclinD1, which can promote the cell cycle from G1phase to S phase transformation [9]. However, we found there was little difference in the cell proportion of G1, G2/M and S phase between UBE2D3 over-expressed cells and control cells, this observation suggested that the cell cycle might not be the key factor in UBE2D3 mediated enhancement of radiosensitivity. However, when exposed to irradiation, majority of tumor cells have cell cycle redistribution. And cell cycle arrested slightly in G2/M phase allows less time to repair damage thus confer radiation sensitivity [18]. Cellular radiosensitivity can be predicted from the features of the cell cycle redistribution [19]. We placed cells under the linear accelerator with 6 Gy X-ray, which was followed by cell cycle detection. As expected, the proportion of G2/M phase in UBE2D3 over-expressed cells was less than that in the control cells. In contrast, the G1 phase was longer in UBE2D3 over-expressed cells than the control cells.

The cell cycle arrest after IR could be due to DNA damage repair [20]. Cellular responses to DNA damage are coordinated primarily by the ATM-Chk2 and ATR-Chk1 pathways, which are activated by DNA double-strand breaks (DSBs) and single-stranded DNA respectively [21]. DSBs is the fundamental mechanism of tumor radiotherapy [22]. Therefore, how cells response to DNA damage is associated with radiosensitivity of tumor cells. A number of proteins are involved in these pathways [23]. We examined several proteins involved in them, showing UBE2D3 over-expressed cells expressed lower levels of these proteins than the control cells after IR. These findings suggested that UBE2D3 suppresses DNA damage response by downregulating the expression of the key proteins in the DNA damage response pathways. Because these proteins play a central role in cell-cycle regulation, transmitting DNA damage signals to downstream effectors of cell-cycle progression. It could be explained that the cell cycle arrested mentioned above. These proteins are also major components in the head of classical DNA damage repair pathway, giving us sufficient reason to believe that the enhanced radiosensitivity of UBE2D3 over-expressed cells is caused by the decreasing capacity of DNA damage repair.

The less DNA damage was repaired, the more DNA damage foci could be observed. The number of radio-induced DSBs is closely correlated with the number of histone gamma-H2AX (γ-H2AX) foci [24]. We found that UBE2D3 overexpression had little impact on DNA damage foci until exposed to X-ray. It indicates that UBE2D3 had little impact on DNA damage foci. UBE2D3 overexpression decreased DNA damage repair capacity thus resulted in the improved DNA damage foci after IR.

Our previous study showed that telomerase and its subunit hTERT were negatively correlated with radiosensitivity of the cancer cells [3, 7, 15, 16]. hTERT is a ribonucleoprotein enzyme essential for the replication of chromosome termini in most eukaryotes [22]. In this study, we observed the hTERT mRNA levels were increased significantly, while the protein levels of hTERT were decreased in UBE2D3 over-expressed cells. This difference in mRNA and protein levels could be due to the degradation of hTERT protein, and then the corresponding negative feedback pathway in the cells eventually led to the increase of mRNA hTERT. Studies by others have shown that hTERT could be modified by ubiquitin [25], leading to proteasomal degradation [26], and UBE2D3 is the key factor of ubiquitin proteasome pathway [27]. Therefore, we hypothesize that UBE2D3 can degraded hTERT through the ubiquitin pathway. This hypothesis was validated by the observation that the treatment UBE2D3 overexpressing cells with the the protesome inhibitor (MG132) resulted in higher levels of ubiquitined hTERT than the control cells treated with the inhibitor. The levels of ubiquitined hTERT protein were very low in both UBE2D3 over-expressed cells and control cells without MG132 treatment. This finding suggested that the UBE2D3 had the ability to stimulate hTERT degradation by ubiquitin-dependent proteolysis. There was no significant difference in hTERT expression level after MG132 interferation in the two cell lines, which proved that UBE2D3 was really involved in the process of hTERT ubiquitined degradation.

We previously documented that the telomerase activity was correlated with cancer cell's radiosensitivity [7]. Telomerase is active in progenitor and cancer cells, but inactive, or very low activity in normal somatic cells [28]. The primary function of hTERT is to elongate telomeres by acting as a reverse transcriptase that adds simple sequence repeats to chromosome ends [29]. We found there was significant decrease in both telomerase activity and relative telomere length in the UBE2D3 overexpressing cells. This might be due to low expression level of hTERT decreased telomerase activity thereby blocked telomere lengthening, eventually caused radiosensitive.

We conformed the *in vitro* observation by the *in vivo* studies, showing that without irradiation, tumor growth rate of mice in OE group was slower than that of the animals in NC group. The tumors in mice of OE group were rapidly shrunk and even disappeared completely by radiotherapy, while the tumors in mice of NC group remained unchanged. These results confirmed UBE2D3 enhance radiosensitivity *in vivo* and *in vitro*. Immunohistochemical result showed that hTERT expression in tissues of OE mice was lower than that of NC mice, suggesting that UBE2D3 enhanced the *in vivo* radiosensitivity also through the hTERT degradation. Because there was little difference in the body weight between mice in the two groups, it is unlikely that nutrition could contribute to tumor growth. These findings indicated that to inhibit hTERT expression is a reasonable approach for enhancing UBE2D3-mediated radiosensitivity of EsC. However, further research should be needed to demonstrate the improving efficacy of EsC radiotherapy.

In conclusion, we have provided both *in vitro* and *in vivo* evidence to support the role of UBE2D3 in enhancing the cellular radiosensitivity of Esc cells. UBE2D3 degraded hTERT and enhanced radiosensitivity of Esc cells through ubiquitin-proteasome pathway.

These data are critical not only for our understanding of UBE2D3-mediated enhancement of radiosensitivity in cancer cells but also for the design and development of UBE2D3-based radiotherapy for EsC.

## MATERIALS AND METHODS

### Cell culture

EC109 cells were cultured in RPMI1640 supplemented with 10% FBS (Hyclone) in 5% CO_2_ at 37°C. EC109 cells were transfected with the expression plasmid encoding UBE2D3, and stable transfectants were subsequently established.

### Reverse transcriptase-polymerase chain reaction analysis

Total RNA was isolated from cells using TRIzol Reagent (MRC Inc., Cincinnati, Ohio), and first-strand cDNA was synthesized using the Revert Aid First Strand cDNA Synthesis Kit (Fermentas Inc., Hanover, Md). The UBE2D3 cDNA was amplified by two steps (60 minute at 42°C, 5 minute at 70°C) using the primers 5′-CCGGACCTTTGAGCATACAC-3′ (forward) and 5′-GCCTTGATATGGGCTGTCAT-3′ (reverse). The amplification of GAPDH cDNA was selected as an internal control. The cycle for PCR used was as follows: 40 cycles of 94°C for 30 s, 60°C for 30 s and 72°C for 30 s. All experiments were repeated at least 3 times.

### Western blot

Cell pellets were lysed in RIPA buffer and immunoblot analysis was performed using standard methods. The following antibodies were used: CHK1 (Proteintech, 10362-1-AP), CHK2 (Proteintech, 13954-1-AP), GAPDH (Abcam, ab37168), ATM (CST, #2873), ATR (CST, #2790), hTERT (santa cruz, sc7241), cyclin D1 (bioworld, BS1741), UBE2D3 (Proteintech, 11677-1-AP), ubiquitin (CST, #3936), γH2Ax (Abcam, ab11174), BRCA1 (Proteintech, 20649-1-AP).

### Clonogenic assay

For assessment of UBE2D3, EC109 cells were plated at low density in 9.6 cm^2^ flasks, and cultured over night. Cells were then irradiated with doses of 0 to 10 Gy of X-rays and then cultured under standard conditions for 14 days. After fixation and staining with 1% (weight/volume) crystal violet (Sigma) in dehydrated alcohol, colonies of > 50 cells were scored, and surviving fractions were normalized for the plating efficiency of mock-irradiated cells. Survival curves were analyzed according to the multitarget-single hit models.

### Cell cycle assay

The cell cycle was assessed in the cells with or without 6 Gy X-rays disposing, and then cultured for the indicated times. Cells were fixed and then treated with RNase for 20 min before addition of 5 mg/ml propidium iodide followed by flow cytometry (Beckman Coulter, Brea, CA, USA) analysis.

### Immunofluorescence

Cells with or without X-ray treatment were fixed with 4% formaldehyde for 15 min and permeabilized with 0.2% Triton X-100 in PBS for 10 min at room temperature. Cells were then incubated with the primary antibody overnight at 4°C, washed and incubated with the secondary antibody. Cell nuclei were stained with DAPI (Sigma, USA). Fluorescence was observed using a confocal microscope (Carl Zeiss LSM710, Germany).

### PCR-ELISA assay

Protein concentrations were determined by the BSA assay. The telomerase activity of each sample was determined by the Telo-TAGGG Telomerase PCR-ELISA kit (Roche, Basel, Switzerland). The absorbance of each sample was determined at 450 nm using a microplate reader (Bio-Rad, Hercules, CA, USA) (with a blank reference wavelength of 690 nm) 30 min after addition of the stop reagent. Data were normalized using the renilla luciferase assay. Each experiment was performed three times in triplicate wells.

### Co-immunoprecipitation

The protein for co-immunoprecipitation were collected using a Universal Magnetic Co-IP Kit (Active Motif, CA, USA). To confirm the interaction of endogenous proteins, lysates were precleared with 25 μL Anti-Rabbit IgG Magnetic Beads (Active Motif) for 1 h at 4°C. The supernatant was discarded, and anti-hTERT antibody were incubated with the precleared cell lysates for 4 h at 4°C. The Magnetic Anti-Rabbit IgG Bead complexes were washed three times with IP wash buffer (Active Motif) and eluted in 2 × SDS loading buffer, followed by SDS/PAGE and immunoblotting.

### Xenograft tumors in nude mice

Mice were purchased from Hunan SJA Laboratory Animal Co., Ltd, Changsha, Hunan, and were handled in accordance with the Novartis Institutes for BioMedical Research (NIBR) Animal Care and Use Committee protocols and regulations. To detect the *in vivo* effects of UBE2D3 on radiosensitivity, we selected the stable cell lines (EC109-pEGFP cells and EC109-pEGFP-UBE2D3 cells) to generate xenograft mouse tumor model. Briefly, EC109-pEGFP cells or EC109-pEGFP-UBE2D3 cells were subcutaneously injected into the right dorsal leg of BALB/c athymic nude mice (aged 4 to 6 weeks) which were named as NC and OE group respectively (Department of Laboratory Animals, Zhongnan Hospital of Wuhan University). Each group had 10 mice (half the male and female). The animal experiments were approved by the Institutional Animal Care and Use Committee of Wuhan University and performed following Institutional Guidelines and Protocols. The body weight of mice, longest diameter “a” and the shortest diameter “b” of tumors were measured every 3 days and the tumor volume was calculated with the following formula: tumor volume (in mm^3^) = a × b^2^ × 0.5 [30]. When the volume of tumors reached 0.5 to 1.0cm in diameter (about 20 days post injection), the mice were exposed to 10 Gy X-ray once every 6 days for a total of two exposures. Applicator sized of 15 × 15 cm, the final radiation field for tumor was expanding 1 cm around the tumor edge with lead blocks shielding, covering 1 cm bonus on the surface of tumor to enhance subcutaneous radiation dose, source skin distance(SSD) was 100 cm, and 9 MeV electron beam IR was used. At the end of the study, animals were sacrificed by cervical dislocation, and tumor tissues were excised for immunohistochemical analysis and snap frozen in liquid nitrogen for biomarker analysis.

### Immunohistochemical analysis

Immunohistochemical staining was performed using the streptavidin-biotin method to detect hTERT (cat no., ab32020; dilution, 1:100; Abcam, Cambridge, MA, USA) protein expression level.

### Statistics

Except noted otherwise the data are presented as mean ± standard deviations. *P*-values were calculated using a two-tailed *t*-test. *P* < 0.05 is considered significant by *t*-test. SPSS22.0 and Graphpad Prism 5 software were used for the statistical analyses.
